# Knowledge–behavior mechanisms and configurations for drowning prevention among Chinese school-aged children: a hybrid multi-analytical study combining regression-based mediation and fsQCA

**DOI:** 10.3389/fpsyg.2026.1923831

**Published:** 2026-09-14

**Authors:** Changlin Yuan, Xi Yang, Zhiping Li, Shuaifeng Qiu, Siyang Wu, Zhe Xiao, Hui Zhang, Chao Xie

**Affiliations:** School of Physical Education, Hubei Minzu University, Enshi, Hubei, China

**Keywords:** high-risk swimming behaviors, risk attitude, risk perception, self-efficacy, sensation seeking, water safety knowledge

## Abstract

**Objective:**

This study examined the psychological mechanisms and configurational pathways by which water safety knowledge is associated with high-risk swimming behaviors among fifth- and sixth-grade primary school children in Hubei Province, China, with the goal of establishing a theoretical and empirical foundation for targeted drowning-prevention interventions.

**Methods:**

In a hybrid multi-analytical study combining regression-based mediation and fsQCA, we analyzed a single quantitative survey dataset of 1,573 fifth- and sixth-grade primary school children from four schools in Hubei Province, China mean age 11.31 ± 0.46 years; 850 boys (54.04%), 723 girls (45.96%). Data collection employed the self-report Water Safety Knowledge Scale (perceived mastery), Risk Perception Scale, Drowning Risk Attitude Scale, Swimming Self-Efficacy Scale, Sensation Seeking Scale, and Swimming High-Risk Behavior Scale. Regression-based mediation was tested with the PROCESS 4.2 macro (model 82, 5000 bias-corrected bootstrap resamples) in SPSS 27.0, and configurational analysis was conducted in fsQCA 4.2.

**Results:**

(1) After controlling for the grade factor, water safety knowledge had no significant direct effect on high-risk swimming behaviors (β = 0.006, *t* = 0.570, *p* > 0.05). (2) Bootstrap mediation analysis produced a total effect of *ES* = −0.037 (*t* = −2.724, *p* < 0.01, 95% *CI* [−0.059, −0.009]). The direct effect was *ES* = 0.006 (*t* = 0.570, *p* > 0.05, 95% *CI* [−0.014, 0.026]). The mediating effect of risk attitude was *ES* = −0.035 (95% *CI* [−0.050, −0.020]). The mediating effect of self-efficacy was *ES* = 0.002 (95% *CI* [0.00001, 0.005]). The mediating effect of sensation seeking was *ES* = −0.016 (95% *CI* [−0.023, −0.009]). The chain mediating effect of self-efficacy and sensation seeking was significant (*ES* = 0.003, 95% *CI* [0.001, 0.005]). (3) Configurational analysis of high-risk swimming behaviors yielded an overall solution consistency of 0.887 and coverage of 0.431. The solution yielded seven sufficient configurations; the two configurations with water safety knowledge as a core present condition are foregrounded: the “disconnect between knowledge and action—attitude deviation” type and the “disconnect between knowledge and action—impulse breakthrough” type.

**Conclusions:**

(1) Water safety knowledge is associated with high-risk swimming behaviors through two parallel mediating paths: “cognition - emotion” and “motivation - impulse”. (2) Configuration analysis shows that the association between water safety knowledge and high-risk swimming behaviors follows a nonlinear, equifinal, and conjunctural pattern. Drowning-prevention interventions for school-age children may benefit from extending beyond knowledge transmission to comprehensive psychological adjustment.

## 1 Introduction

Drowning is a major global public health problem and a leading threat to child safety (World Health Organization, 2026). Chinese survey data indicate that drowning accounts for 33% of all accidental fatalities each year, and that child drownings comprise over 40% of total drowning incidents (Pengpai News, 2026). Drowning causes high rates of death and disability, which significantly affect the health of school-age children, their families, and society. In China, a primary factor driving the high incidence of drowning among school-age children is the prevalent discrepancy between knowledge and behavior in water-related activities, commonly described as “high knowledge but low practice.” Research shows that although water safety education is widely disseminated among Chinese school-age children, it has not effectively reduced high-risk swimming behaviors (Abadi et al., 2025). In surveys of drowning prevention and related research in China, over 90% of participants in water-related activities demonstrate basic knowledge of drowning prevention. Nevertheless, some children continue to engage in unsafe water activities and dangerous behaviors. This pattern is characterized as the coexistence of high knowledge and persistently high-risk behavior. (Zhong et al., 2026). The simple linear transmission model (K → A → P) assumed by Knowledge, Attitude, and Practice (KAP) research has limitations in explaining the coexistence of high knowledge and high-risk behavior. Knowledge, attitude, and behavior do not necessarily relate causally, and individuals often display “high knowledge but low practice,” a pattern that can contribute to drowning accidents (Carrillo-González, 2025). The core explanation is that, during the school-age years, maturation of the prefrontal “control system” lags behind that of the limbic “reward system.” The reward system is especially sensitive in this period, which amplifies children's approach motivation toward novel stimuli and increases sensation-seeking. At the same time, the prefrontal cortex, as the control region, develops more slowly, producing a temporary deficit in impulse inhibition (Shulman et al., 2016). Additionally, rising hormone levels during school age further heighten the limbic reward system's sensitivity, producing a sharp increase in motivation driven by peer recognition and novel, stimulating experiences. The uneven maturation of the cortico-limbic dopamine system reduces the regulatory efficiency of control regions in the brain (Poon et al., 2019; Fryt, 2017). Multiple overlapping influences make school-age children more likely to seek immediate stimulation and ignore long-term safety when they encounter water, producing a gap between cognitive judgment and behavioral execution. The developmental characteristics of this stage mean that even children with intact risk-assessment skills can display a persistent execution gap—“high knowledge but low action”—under strong emotional arousal or peer pressure. Developmental research indicates that cognitive control and reward sensitivity follow divergent trajectories through childhood and adolescence (Shulman et al., 2016; Steinberg et al., 2008). This literature motivates our focus on preadolescent children, the stage preceding the mid-adolescent rise in sensation seeking and the stage at which systematic water-safety instruction occurs; the present design does not test developmental hypotheses directly. This study integrates the Knowledge, Attitude and Practice (KAP) model with the Health Belief Model (HBM), combining the KAP model's linear logic that emphasizes knowledge and attitudes with the HBM's decision-making framework that evaluates perceived threat, benefits and barriers, and self-efficacy (Moran, 2007; Jones et al., 2015). We incorporated school-age children's personality traits, such as sensation seeking, as constraints on belief evaluation to account for the gap between knowledge and behavior (high knowledge but low practice) in water safety. Considering the neurodevelopmental profile of this age group, the study highlights that internal factors—cognitive appraisal and emotional arousal—and external cues, such as peer contexts and action prompts, jointly shape behavioral decisions, offering a theoretical framework for the complex psychological mechanisms underlying drowning prevention among Chinese school-age children.

### 1.1 The relationship between water safety knowledge and high-risk swimming behaviors

Water Safety Knowledge (WSK) denotes the knowledge and understanding individuals use to protect themselves and others in water environments (Guy et al., 2024). Studies indicate that groups with higher WSK are less likely to engage in high-risk behaviors than groups with lower WSK (Shi et al., 2025). WSK enables children to recognize warning signs more reliably, guides them away from hazardous actions, and fosters adherence to relevant safety norms. It also helps children make initial assessments of their health, weather, and water quality, and it equips them to take immediate, potentially life-saving actions (Luo et al., 2024; Tan et al., 2025). According to the International Life Saving Federation (ILS), intervention through water safety education is widely regarded as an effective preventive measure against drowning. High-risk Swimming Behaviors (HSB) denote actions or choices during swimming and other water activities that substantially raise an individual's likelihood of drowning or sustaining injury. Such behaviors commonly stem from poor risk perception, thrill-seeking, lack of self-discipline, or deliberate breaches of safety rules (Ostrowski et al., 2024). High-risk swimming behaviors include ignoring water-safety rules and warning signs, overestimating one's abilities while underestimating potential dangers, lacking self-rescue skills, and yielding to peer pressure. These behaviors also serve as external indicators of a “knowledge–action gap” among school-age children in drowning incidents, collectively reflecting the cognitive–behavioral separation of “knowing but finding it difficult to act” at this stage (Luo and Mei, 2025). Research indicates that adequate water-safety knowledge enables individuals to reduce high-risk swimming behaviors (Moran, 2007). However, school-age children's behavior is not governed solely by knowledge; it is more closely linked to deeper psychological mechanisms and physiological regulation (Luo et al., 2024; Ostrowski et al., 2024; Cho and Lee, 2005). In an empirical study on risk perception and attitudes toward drowning risk, water safety knowledge has a significant negative prediction of drowning risk behaviors, which reflects the “buffering” effect of knowledge on high-risk behaviors (Luo et al., 2024). The moderated mediation analysis also shows that water safety knowledge significantly and negatively predicts high-risk swimming behaviors (Shi et al., 2025). Accordingly, this study proposes **Hypothesis H1**: Water safety knowledge negatively predicts high-risk swimming behaviors among school-age children.

### 1.2 The chained mediating effect of risk perception and risk attitude

Risk perception (RP) denotes an individual's attitude toward hazards and their assessment of the likelihood and potential consequences of those hazards (Cho and Lee, 2005). In the Health Belief Model (HBM), RP constitutes the initial cognitive driver of behavioral decision-making and comprises perceived susceptibility and perceived severity. Its primary role is to identify the “risk problem” posed by health threats. Evidence shows that the conversion of safety knowledge into safety behavior is not direct; rather, it is mediated by multiple cognitive and emotional mechanisms through risk perception (Luo et al., 2024). The dual-process theory divides human decision making into an intuitive, fast, and automatic “System 1” and a deliberate, slow, and analytical “System 2.” In school-age children, the imbalance between cognitive control and emotion–reward systems manifests as a greater reliance on System 1. Such intuition-dominated decision making reduces the accuracy and timeliness of risk perception and assessment, producing distorted judgments of threat urgency that can precipitate high-risk behaviors (Shulman et al., 2016; Kahneman et al., 2011). The research found that adolescents' perception of drowning risk functions as a protective factor, and that perceived susceptibility and severity substantially attenuate the adverse effects of peer risk-taking behaviors (Luo and Mei, 2025). Empirical studies grounded in the risk-protection integration framework and KAP (Knowledge-Attitude-Practice) theory indicate that increased water safety knowledge effectively elevates risk perception and thereby suppresses drowning-risk behaviors (Luo et al., 2024). Building on these findings, this study proposes **Hypothesis H2**: risk perception mediates the relationship between water safety knowledge and high-risk swimming behaviors.

Risk attitude (RA) denotes an individual's propensity to accept or avoid risk in uncertain situations, and it directly influences the occurrence of dangerous behaviors (Pan et al., 2020). Within the KAP framework, attitude occupies the central role in the “knowledge → belief → behavior” transmission chain (Moran, 2007). Empirical work across multiple fields supports this theory: health attitudes exert a stronger influence on risky behaviors than knowledge alone, and they are pivotal in converting knowledge into action (Alves, 2024). Systematic reviews and meta-analyses likewise identify attitude as a key mediator between health literacy and actual behavior (McAnally and Hagger, 2023). Attitudes can influence behavior more effectively than mere knowledge because they operate through a rational analysis and decision-making model. This model internalizes objective information into stable behavioral tendencies by weighing values and integrating preferences (Kahneman et al., 2011). Empirical work shows that increasing knowledge enables individuals to develop positive beliefs and attitudes, which in turn promote healthy behaviors (Alves, 2024). Knowledge must be coupled with a positive attitude to effectively suppress risky behavior. Merely increasing knowledge has only a modest direct impact on reducing such behaviors (Alves, 2024). Sufficient water-safety knowledge fosters a positive safety attitude, which in turn lowers the likelihood of high-risk behavior in aquatic settings. Water-safety knowledge can therefore influence high-risk swimming behavior indirectly via the mediating role of drowning-risk attitude (Luo et al., 2024). Accordingly, we propose **Hypothesis H3**: Risk attitude mediates the relationship between water-safety knowledge and high-risk swimming behaviors.

Research based on the integrated theoretical model of the Knowledge, Attitude, and Practice (KAP) theory and the Health Belief Model (HBM) shows that the transformation from knowledge to behavior is not a direct prediction of the threat recognition by risk perception. It may also involve the transfer from cognitive evaluation to behavioral tendency through risk attitude (Liao and Yang, 2023). Luo et al. found that water safety knowledge predicts drowning-risk behaviors via three indirect pathways. The chained mediator linking risk perception to risk attitude represented the second-largest pathway, exceeded only by the single mediation of risk perception. Overall, the three indirect paths accounted for 41.5% of the total effect (Luo et al., 2024). The chained mediation from risk perception to risk attitude describes a full psychological pathway from “knowledge” to “action.” Knowledge first triggers an immediate appraisal of health threats via “System 1.” Then, through deliberative evaluation by “System 2,” a stable attitudinal disposition emerges, which ultimately guides behavior. The chain-mediating effect linking risk perception to risk attitude has shown strong robustness in studies of risk-protective behaviors (Thompson and Hansen, 2013). Drawing on this framework, this study proposes **Hypothesis H4:** Risk perception and risk attitude jointly mediate the relationship between water safety knowledge and high-risk swimming behaviors.

### 1.3 The chain mediating effect of self-efficacy and sensation seeking

Self-efficacy (SE) denotes an individual's belief in their capacity to perform specific behaviors successfully (Tsang et al., 2012). SE not only directly predicts behavioral intention but also influences behavioral change indirectly through four processes: cognitive, motivational, emotional, and selective (Jones et al., 2015). Recent scholarship treats SE not only as an antecedent of behavior change but also as a key psychological mediator linking intention to action. SE partially mediates the relationship between water-safety skills and high-risk swimming behaviors (Tan et al., 2025). Zhang et al. found that self-efficacy mediates the relationship between risk perception and health-protective behaviors, and that increases in efficacy beliefs promote such behaviors (Zhang et al., 2022). In the evaluation of the water-safety education program “Eyes Save Lives,” structured knowledge interventions improved parents‘ and caregivers' water-safety behaviors, attitudes, and self-efficacy simultaneously, reflecting an empowerment effect in which knowledge accumulation raises efficacy beliefs and thereby encourages healthier behavior (Love-Smith et al., 2022). Prior work has proposed that self-efficacy can act as a “double-edged sword” (Tan et al., 2025). When efficacy beliefs exceed a reasonable calibration threshold and shift toward overconfidence as knowledge accumulates, they become an “illusion of control” or “confidence inflation.” At that point, efficacy beliefs no longer reflect actual ability and instead trigger risky behavior (Tan et al., 2025). Therefore, this study proposes **Hypothesis H5**: Self-efficacy mediates the relationship between water safety knowledge and high-risk swimming behaviors.

Sensation seeking (SS) denotes a stable personality trait in which individuals pursue novel, complex, and intense sensory experiences and accept physical, social, legal, and financial risks to obtain them (Zheng et al., 2014). As a personality risk factor, sensation seeking contributes to high-risk behaviors and is considered temporally stable and relatively unaffected by external influences (Lynne-Landsman et al., 2011). Research shows that emotion-focused schema therapy and mindfulness-based stress reduction significantly decrease children's sensation seeking (Khabaznoori and Azad, 2024; Najafi Chaleshtori et al., 2022). In studies of dangerous driving, sensation seeking correlates positively with risky driving behavior. These findings suggest that safety interventions should lower dangerous-driving tendencies by targeting mediating variables—specifically by reducing sensation seeking and strengthening safety attitudes—and that such interventions should begin in the school-age years (Hassan et al., 2017). A high degree of sensation seeking can markedly delay individuals' responses to hidden hazards, leading them to neglect necessary coping measures and thereby increasing the occurrence of high-risk swimming behaviors (Zhang et al., 2023). Shi et al. were the first in water safety research to establish a moderated mediation model that treats sensation seeking as a mediator, showing that greater knowledge can suppress sensation seeking and reduce high-risk behaviors (Shi et al., 2025). Accordingly, this study proposes **Hypothesis H6**: Sensation seeking mediates the relationship between water safety knowledge and high-risk swimming behaviors.

In the KAP-HBM integrated model, the HBM assumes that individuals act as rational information processors who make health decisions by weighing benefits against costs. Yet an inconsistency arises: some individuals with high knowledge still engage in high-risk swimming behavior. To account for this, the model is extended to a “cognition–emotion–personality” framework. Evidence indicates that the behavior of school-age children and adolescents is driven more strongly by emotional processes and personality traits than by purely rational cognitive calculations (Shi et al., 2025). Previous studies show that self-efficacy and sensation seeking each partially mediate the translation of skills and knowledge into behavior (Shi et al., 2025; Tan et al., 2025). The dual nature of self-efficacy implies that confidence acquired through knowledge accumulation has been proposed to become overconfidence, which in turn may heighten sensation-seeking and prompt more stimulating swimming behaviors (Tan et al., 2025). In extreme sports research (for example, rock climbing, skydiving, and surfing), an “efficacy–impulse” synergistic mechanism holds that efficacy beliefs derived from advanced skills reduce sensitivity to perceived danger and lead individuals to believe they can manage risks. As a result, these beliefs free an impulsive tendency to pursue high-difficulty challenges (Zhang et al., 2023; Hu et al., 2021). Based on these arguments, this study proposes **Hypothesis H7:** self-efficacy and sensation seeking jointly mediate the relationship between water safety knowledge and high-risk swimming behaviors.

The theoretical hypothesis model of this study is shown in Figure 1.

**Figure 1:**
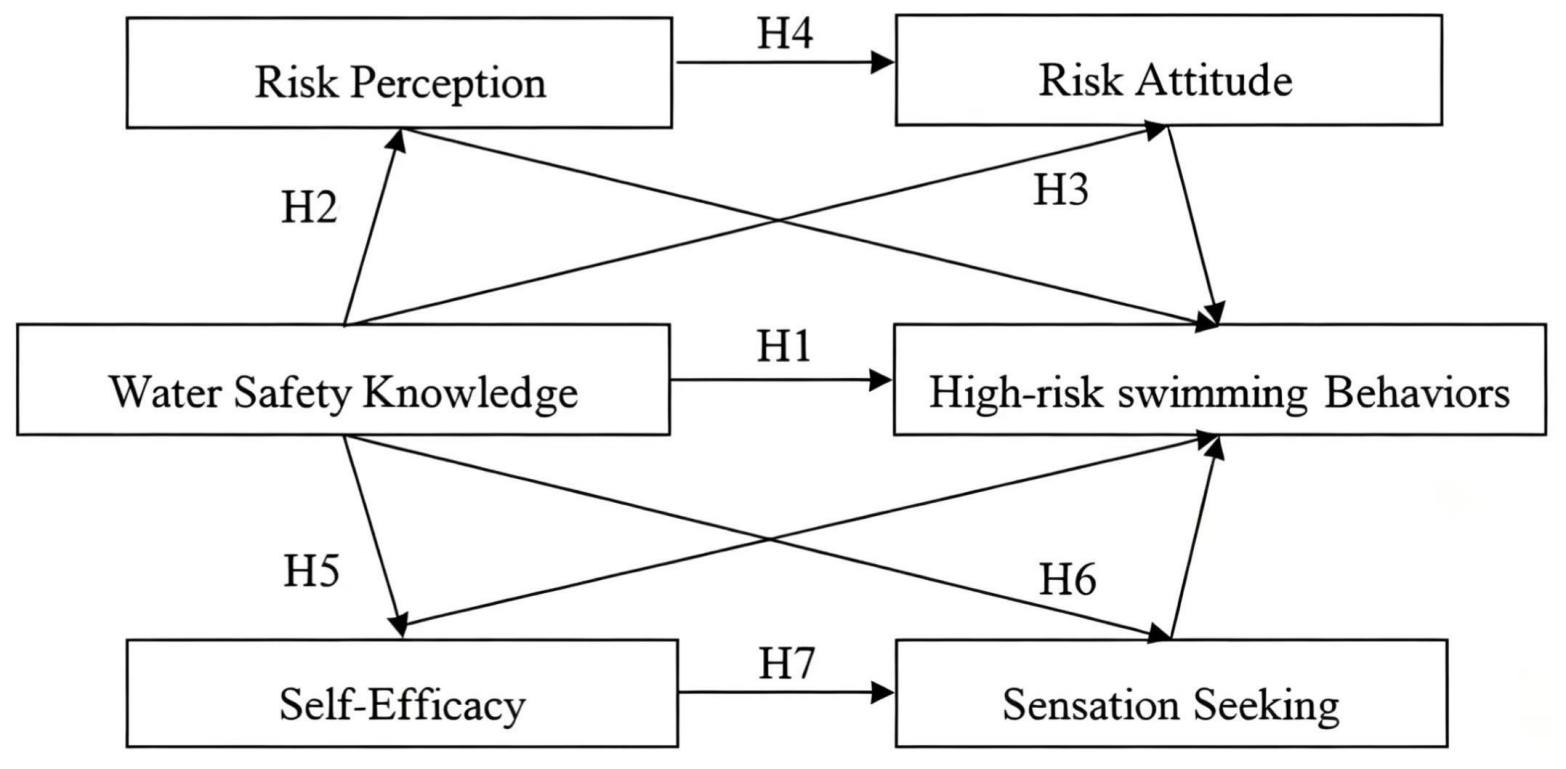
The hypothesized model.

## 2 Methods

**Study design**. This study is a hybrid multi-analytical study combining regression-based mediation and fuzzy-set qualitative comparative analysis (fsQCA). All evidence derives from a single quantitative questionnaire dataset, and no separate qualitative data collection or qualitative coding was performed. Methodological mixing in this study occurs at the level of analytical logic rather than data type. Under one theoretical model, the two approaches answer complementary questions. The symmetric, regression-based mediation analysis estimates the average net effect of each hypothesized pathway from water safety knowledge to high-risk swimming behaviors. The asymmetric, set-theoretic fsQCA identifies combinations of conditions that are sufficient for high-risk swimming behaviors and tests whether the pathways identified by the net-effect model recur as equifinal configurations across cases.

### 2.1 Research subjects

We used cluster sampling to survey primary school children in Hubei Province, China. In 2025, we conducted school-level cluster sampling at four public primary schools in Hubei Province and invited all fifth- and sixth-grade students in the participating classes. Eligible students were enrolled in grade five or six, gave their own assent, and had written consent from a parent or legal guardian. Of 1,800 distributed questionnaires, 227 were excluded, comprising 135 with excessive missing data and 92 with uniform response patterns, which left 1,573 valid cases (87.4%). The four schools (anonymized as Schools one to four) contributed 492, 161, 595, and 325 cases, respectively. Participants were in fifth grade (*n* = 999) and sixth grade (*n* = 574), with a mean age of 11.31 ± 0.46 years. Gender was coded as 1 = male and 2 = female; the sample included 850 boys (54.04%) and 723 girls (45.96%) (Table 1). Because students were nested within schools, we calculated the intraclass correlation coefficient (*ICC*) for the outcome variable at the school level. The ICC indicated negligible school-level dependency (*ICC* = 0.015). As a sensitivity analysis, all models were re-estimated with school fixed effects, and the pattern of significant and non-significant paths was unchanged in all five equations (path coefficients changed by at most 0.017); the reported models therefore treat observations as independent.

**Table 1:** Demographic characteristics of the sample (*N* = 1,573).

Characteristic	Category	*N*	%
Gender	Boy	850	54.04
	Girl	723	45.96
Grade	Fifth	999	63.51
	Sixth	574	36.49
School	School 1	492	31.28
	School 2	161	10.24
	School 3	595	37.83
	School 4	325	20.66
Age, years	*M* = 11.31	*SD* = 0.46	

In line with the Declaration of Helsinki, informed consent was obtained from all participants and their legal guardians prior to data collection, and personal information was safeguarded. The study was reviewed and approved by the Ethics Committee of Hubei Minzu University [No. (2024)101].

### 2.2 Measurement tools

#### 2.2.1 Water safety knowledge scale

The Student Water Safety Knowledge Scale developed by Xia (Xia et al., 2012) was adopted to assess children's self-assessed mastery of water safety knowledge. This scale consists of nine items, covering content such as safety signs, safety common sense, and lifeguarding procedures (sample items: “knowledge of water safety signs,” “knowledge of self-rescue in the water,” and “first-aid measures during floods”). Respondents rate each statement on agreement anchors, so the instrument measures self-assessed knowledge mastery—that is, children's perceived familiarity with water safety content—rather than objectively tested knowledge. The original scale was developed and validated in two Chinese samples (pilot test *N* = 495; formal test *N* = 2,554), with items screened by exploratory factor analysis, content validity assessed by the content validity ratio, and structural validity assessed by exploratory and confirmatory factor analyses (*KMO* = 0.911; four factors each explaining approximately 10.2%−10.9% of the variance, cumulatively 42.305%). The scale is scored using a five-point Likert scale, where one represents “completely inconsistent” and five represents “completely consistent”. A higher total score indicates a better mastery of water safety knowledge. The Cronbach's α coefficient of this sub-scale is 0.943, indicating good reliability. The composite reliability (*CR*) is 0.955, and the average variance extracted (*AVE*) is 0.704. In the current sample, the Cronbach's α coefficient is 0.955.

#### 2.2.2 Drowning risk attitude scale

The Drowning Risk Attitude Scale (DRAS) is a standardized assessment tool used to measure individuals' cognitive, judgmental, and behavioral tendencies toward drowning-related risks (Xia et al., 2012). The Chinese version of the DRAS has undergone reliability and validity tests, demonstrating good repeatability, structural validity, and content validity, and is suitable for Chinese children and adolescents. The scale consists of a total of 10 items and uses a five-point Likert scale, ranging from one point (strongly disagree) to five points (strongly agree). The scores of all items are averaged to obtain the composite score. A higher total score indicates weaker awareness of drowning risks and a stronger willingness to engage in risky behaviors. The scale has good internal consistency, with a Cronbach's α coefficient of 0.913, indicating good reliability and the ability to stably assess an individual's attitude toward drowning risks. The composite reliability (*CR*) is 0.940, and the average variance extracted (*AVE*) is 0.614. In the current sample (*F*64–*F*68 reverse-scored + Q69–Q73), the Cronbach's α coefficient is 0.935.

#### 2.2.3 Swimming high-risk behavior scale

The Child and Adolescent Swimming High-risk Behavior Scale developed by our research group was used. The scale consists of four dimensions, namely errors, slips, general violations, and aggressive violations, with a total of 20 items. It can cumulatively explain 72.362% of the total variance. Confirmatory factor analysis indicated that the model fitted well (χ^2^/df = 3.672, *CFI* = 0.938, *NFI* = 0.952, *TLI* = 0.957, *IFI* = 0.938, *RMSEA* = 0.045), suggesting good structural validity. The Cronbach's α coefficient of the total scale was 0.859, and the coefficients of each dimension were around 0.80, indicating good internal consistency reliability. The scale uses a five-point likert scale, where one point means very inconsistent and five points means very consistent. The higher the score, the more likely the occurrence of high-risk swimming behaviors. The composite reliability (*CR*) is 0.934, and the average variance extracted (*AVE*) is 0.438. In the current sample, Cronbach's α was 0.898.

#### 2.2.4 Swimming self-efficacy scale

The Swimming Self-Efficacy Scale developed by Theodorakis was used to measure the perceived intensity of the subjects‘swimming skill levels (Theodorakis, 1995). It measured the subjects' confidence in completing six swims at progressive distances of 20–45 meters within 20 seconds. Each item on the scale was scored from one to 10 (one = completely uncertain, 10 = very certain), and the average score of the six items was taken as the self-efficacy score. A higher score indicated a higher level of self-efficacy. The Cronbach's α coefficient of the original scale was 0.88 to 0.91, indicating good reliability and validity. The composite reliability (*CR*) is 0.949, and the average variance extracted (*AVE*) is 0.758. In the current sample, Cronbach's α was 0.946.

#### 2.2.5 Sensation seeking scale

The Sensation Seeking Scale (Steinberg et al., 2008) was used. Six items assess the preference for novel, exciting, and slightly frightening experiences (e.g., “I like new and exciting experiences, even if they are a little frightening”), rated on a five-point scale (one = does not describe me at all, five = describes me very well) and averaged. The Cronbach's α coefficient of this scale is 0.70, suggesting good reliability. The composite reliability (*CR*) is 0.920 and the average variance extracted (*AVE*) is 0.659. In the current sample, Cronbach's α was 0.917.

#### 2.2.6 Drowning risk perception scale

The Drowning Risk Perception Scale (DRPS), which consists of 14 items, is used to evaluate individuals' levels of drowning risk perception (Laosee et al., 2014). The reliability and validity of the Chinese version of DRPS have been described previously. A five-point Likert scale was used for scoring, ranging from one (strongly disagree) to five (strongly agree). All items were averaged to obtain the composite score, with a higher score indicating a higher perception of drowning risk. In the current sample, the confirmatory factor analysis of the six-factor measurement model including this scale yielded χ^2^/df = 5.957, *CFI* = 0.863, *TLI* = 0.857, and *RMSEA* = 0.056. The composite reliability (*CR*) is 0.858, and the average variance extracted (*AVE*) is 0.305. In the current sample, Cronbach's α was 0.858.

### 2.3 Data processing

All composites were constructed from the raw items under the item-level scoring key, in which each item contributes to exactly one construct. Data organization, cleaning, descriptive statistics, Pearson correlation, and Harman single-factor analysis were performed in SPSS 27.0 (IBM Corp., Armonk, NY, USA). Mediation testing used Hayes's PROCESS macro model 82 (PROCESS 4.2; A. F. Hayes) with the nonparametric bias-corrected bootstrap method and 5000 resamples (Hayes, 2017). Configuration analysis, including case ranking of configurations and reverse case analysis, was conducted in fsQCA 4.2. We calibrated the six measures into fuzzy sets using the direct method. For each measure, the 95th percentile was set as the anchor for full membership, the 50th percentile as the crossover point of maximum ambiguity, and the 5th percentile as the anchor for full non-membership; all anchor values are reported in Table 2. We used sample percentiles because the composite scales have no established external standards for full membership in the underlying theoretical sets, so the anchors locate membership relative to the observed distribution of Chinese school-age children. Two implications are stated explicitly. First, membership scores are sample-relative, and replications in other populations should recalibrate rather than reuse these anchors. Second, each fuzzy score expresses membership in the set of high values of the respective condition, so the crossover point, not the anchor labels, carries the substantive meaning. For risk attitude and sensation seeking, the response distributions are concentrated at the scale minimum, so the 50% and 5% calibration anchors were set at the theoretical points of 2.000 and 1.000, respectively (Table 2); membership for these two conditions is therefore differentiated mainly among values above the minimum, and the robustness of the solution to alternative percentile anchors is reported in the Results. For the truth-table analysis we set the case frequency threshold at 3, the raw consistency threshold at 0.80, and the PRI consistency threshold at 0.65. With five conditions, 32 rows are logically possible; 27 rows met the frequency threshold and covered 99.49% of the 1,573 cases, well above the conventional benchmark of retaining roughly 75 to 80 percent of cases (Ragin, 2008; Schneider and Wagemann, 2012). The 0.80 raw consistency threshold is the conventional lower bound for sufficiency claims (Ragin, 2008), and the 0.65 PRI threshold prevents configurations that are simultaneously subsets of the outcome and of its negation (Schneider and Wagemann, 2012). The cutoff is applied without exception, and any configuration at or below it is excluded rather than reported. We report the intermediate solution (Fiss, 2011), in which logical remainders are incorporated through directional expectations (the presence—that is, the high state—of each condition was expected to contribute to the outcome, rather than its absence), and conditions are classified as core or peripheral according to their presence in the parsimonious and intermediate solutions. Necessity analysis, truth-table construction, and stability testing were then carried out. Because the data are cross-sectional, the configurations describe sufficiency relations among concurrent measures, not temporally or experimentally identified causal effects. We therefore use configurational association and sufficiency language throughout and avoid causal readings of the solution.

**Table 2:** Calibration anchors for fuzzy-set membership.

Percentage	WSK	RP	RA	SE	SS	HSB
95%	5.000	4.785	3.000	7.500	3.000	2.400
50%	3.555	3.285	2.000	1.666	2.000	1.400
5%	1.222	1.714	1.000	1.000	1.000	1.000

We tested the mediation hypotheses with observed composite scores using the PROCESS macro (model 82) rather than a latent structural equation model for four reasons. First, our hypotheses concern specific indirect effects among established composite measures with a covariate, and regression-based mediation with bias-corrected bootstrap confidence intervals (5,000 resamples) is designed for exactly this estimand (Hayes, 2017); for a just-identified observed-variable model such as ours, ordinary least squares and SEM estimation coincide, so the choice reduces to whether measurement error is modeled latently rather than to any difference in point estimation. Second, the observed-variable approach preserves direct comparability with the closest prior studies in this domain, which estimated the same family of indirect paths with the same approach (Luo et al., 2024; Shi et al., 2025; Tan et al., 2025); several of our hypotheses are deliberately confirmatory, and comparability of effect sizes serves that purpose. Third, the constructs show high composite reliability (0.858 to 0.955) and clear discriminant validity (all HTMT below 0.85), which limits, though does not eliminate, the bias that measurement error introduces into observed-variable path estimates. Fourth, the design pairs symmetric net-effect estimation with asymmetric configurational analysis (fsQCA); a latent SEM would address the same net-effect questions but would not alter the configurational arm or the inferential targets. The trade-offs of this choice are acknowledged in the Limitations section.

## 3 Results and analysis

### 3.1 Common method bias test

We employed the Harman's single-factor test to assess the common method bias. The result of the single-factor analysis of all items showed that the proportion of the eigenvalue of the first factor was 24.02%, which was lower than the critical value of 40% (Zhou and Long, 2004). The heterotrait-monotrait ratio (HTMT; Henseler et al., 2015) was used to evaluate the discriminant validity. All HTMT values were below the conservative critical value of 0.85. For details, see Table 3. Convergent and discriminant validity were assessed before the structural models were interpreted. Composite reliabilities ranged from 0.858 to 0.955 and average variance extracted from 0.305 to 0.758; two constructs fell below the 0.50 AVE benchmark (risk perception 0.305, high-risk swimming behaviors 0.438), for which composite reliabilities above 0.60 provide adequate convergent validity following Fornell and Larcker, 1981). The largest HTMT ratio was 0.673. Multicollinearity diagnostics showed variance inflation factors between 1.000 and 1.549, below the conventional threshold of 5 (Hair et al., 2011), indicating that multicollinearity does not threaten the structural estimates.

**Table 3:** Heterotrait-monotrait ratio matrix.

Variable	HTMT	Variable	HTMT
RP-SS	0.152	RA–SE	0.293
RA-SS	0.610	SE–SS	0.325
RP–RA	0.118	SE–HSB	0.245
RP–SE	0.124	SS–HSB	0.516
RP–HSB	0.131	RA-HSB	0.673
WSK–RP/RA/SE/SS/HSB	0.207/0.143/0.125/0.151/0.129	-	-

WSK = Water Safety Knowledge; HSB = High-risk Swimming Behaviors; RP = Risk Perception; RA = Risk Attitude; SE = Self-efficacy; SS = Sensation Seeking

### 3.2 Descriptive statistics and correlation analysis

Table 4 shows that water safety knowledge correlated negatively with risk perception (*r* = −0.189, *p* < 0.01), risk attitude (*r* = −0.134, *p* < 0.01), and sensation seeking (*r* = −0.141, *p* < 0.01), and correlated positively with swimming self-efficacy (*r* = 0.121, *p* < 0.01). Risk perception correlated positively with swimming self-efficacy (*r* = 0.099, *p* < 0.01) and with sensation seeking (*r* = 0.104, *p* < 0.01). Risk attitude showed positive correlations with swimming self-efficacy (*r* = 0.271, *p* < 0.01) and with sensation seeking (*r* = 0.563, *p* < 0.01). Finally, swimming self-efficacy correlated positively with sensation seeking (*r* = 0.299, *p* < 0.01).

**Table 4:** Results of descriptive statistics and correlation analysis.

Variable	*M*	SD	WSK	RP	RA	SE	SS	HSB
WSK	3.459	1.086	1					
RP	3.281	0.852	−0.189^**^	1				
RA	1.315	0.580	−0.134^**^	−0.010	1			
SE	2.762	2.304	0.121^**^	0.099^**^	0.271^**^	1		
SS	1.479	0.764	−0.141^**^	0.104^**^	0.563^**^	0.299^**^	1	
HSB	1.533	0.543	−0.069^**^	−0.016	0.606^**^	0.228^**^	0.467^**^	1

^*^ *p* < 0.05; ^**^ *p* < 0.01. This tables use the same convention.

### 3.3 Mediating effect test

After controlling for grade, we analyzed regression coefficients and the mediating effects of risk perception, risk attitude, sensation-seeking, and self-efficacy on the link between water safety knowledge and high-risk swimming behavior. Water safety knowledge did not significantly predict high-risk swimming behavior (β = 0.006, *t* = 0.570, *p* > 0.05), so Hypothesis H1 was not supported. Water safety knowledge negatively predicted risk perception (β = −0.147, *t* = −7.606, *p* < 0.01), but risk perception did not significantly predict high-risk swimming behavior (β = −0.020, *t* = −1.553, *p* > 0.05), so **Hypothesis H2** was not supported. Water safety knowledge was negatively associated with risk attitude (β = −0.074, *t* = −5.509, *p* < 0.01), and risk attitude was positively associated with high-risk swimming behavior (β = 0.462, *t* = 20.343, *p* < 0.01); thus, **Hypothesis H3** was supported. The effect of risk perception on risk attitude was not significant (β = −0.024, *t* = −1.425, *p* > 0.05), not supporting **Hypothesis H4**. Water safety knowledge was positively associated with self-efficacy (β = 0.257, *t* = 4.846, *p* < 0.01), and self-efficacy was positively associated with high-risk swimming behavior (β = 0.009, *t* = 1.961, *p* < 0.05); therefore, **Hypothesis H5** was supported. Water safety knowledge was negatively associated with sensation-seeking (β = −0.126, *t* = −7.542, *p* < 0.01), while sensation-seeking was positively associated with high-risk swimming behavior (β = 0.129, *t* = 7.417, *p* < 0.01), so **Hypothesis H6** was supported. Finally, self-efficacy was positively associated with sensation-seeking (β = 0.106, *t* = 13.472, *p* < 0.01), supporting **Hypothesis H7**. Regression results are summarized in Table 5 and illustrated in Figure 2.

**Table 5:** Regression analysis.

Variable	***RP***		RA		SE		SS		HSB	
	β	*T*	β	*t*	β	*t*	β	*T*	β	*t*
Grade	−0.001	−0.033	0.034	1.129	0.011	0.093	0.041	1.098	−0.002	−0.115
WSK	−0.147	−7.606^**^	−0.074	−5.509^**^	0.257	4.846^**^	−0.126	−7.542^**^	0.006	0.570
RP			−0.024	−1.425					−0.020	−1.553
RA									0.462	20.343^**^
SE							0.106	13.472^**^	0.009	1.961^*^
SS									0.129	7.417^**^
*R^2^*	0.035		0.019		0.014		0.122		0.393	
*F*	28.928^**^		10.637^**^		11.744^**^		72.780^**^		169.231^**^	

^*^p < 0.05, ^**^ *p* < 0.01.

**Figure 2:**
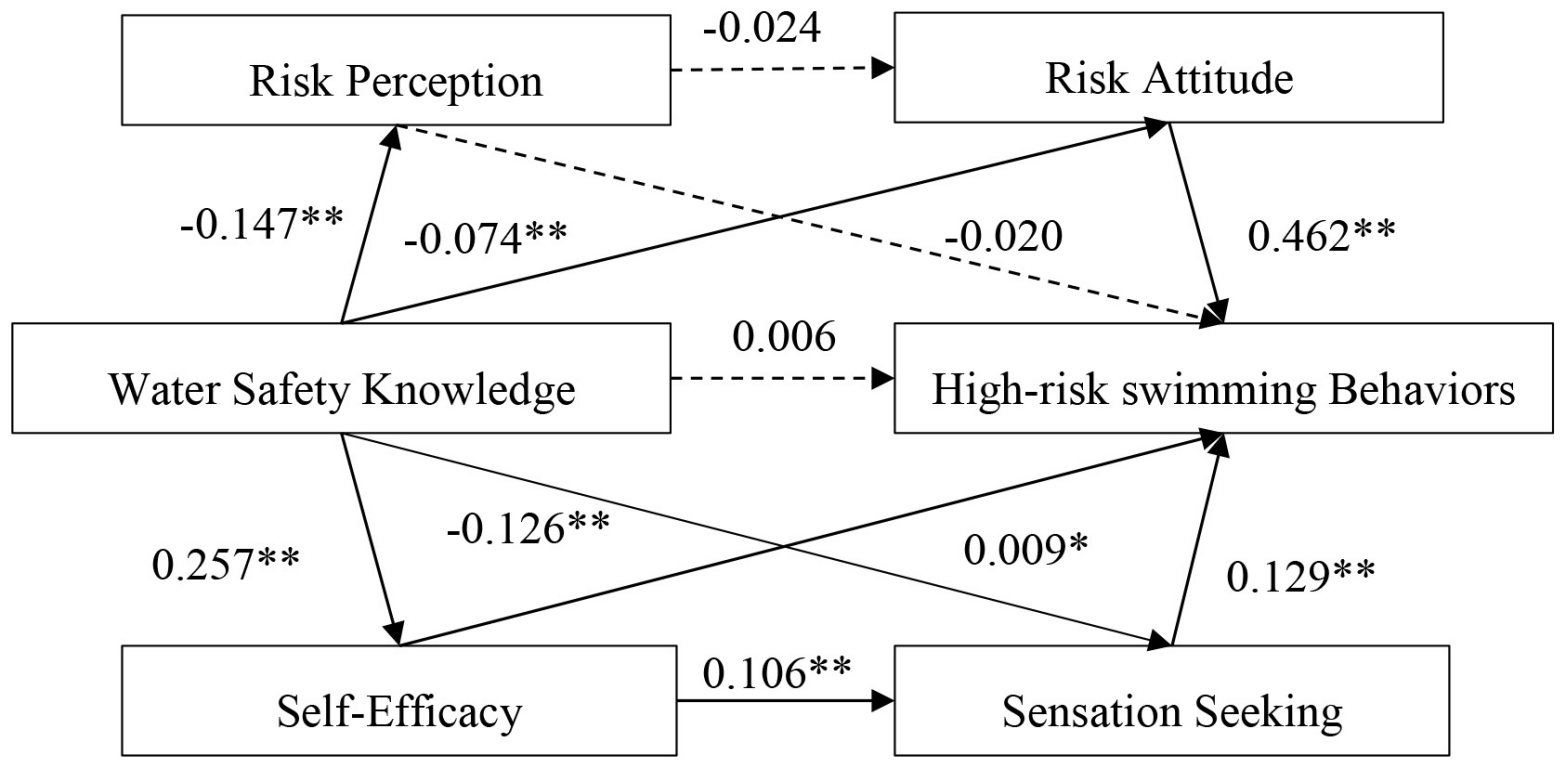
The serial multiple-mediation model (PROCESS model 82).

Bootstrap analysis of mediation showed that the total effect of water safety knowledge on high-risk swimming behaviors was *ES* = −0.037, *t* = −2.724 (*p* < 0.01), 95% *CI* [−0.059, −0.009]. The direct effect of water safety knowledge on high-risk swimming behaviors was not significant (*ES* = 0.006, *t* = 0.570 (*p* > 0.05), 95% *CI* [−0.014, 0.026]), so **hypothesis H1** is not supported. Risk perception did not mediate the relationship between water safety knowledge and high-risk swimming behaviors (*ES* = 0.003, 95% *CI* [−0.001, 0.008]), so **hypothesis H2** is not supported. Risk attitude mediated the relationship between water safety knowledge and high-risk swimming behaviors (*ES* = −0.035, 95% *CI* [−0.050, −0.020]), supporting **hypothesis H3**. The chained mediation of risk perception and risk attitude between water safety knowledge and high-risk swimming behaviors was not significant (*ES* = 0.002, 95% *CI* [−0.001, 0.004]), so **hypothesis H4** was not supported. Self-efficacy mediated the relationship between water safety knowledge and high-risk swimming behaviors (*ES* = 0.002, 95% *CI* [0.00001, 0.005]), supporting **hypothesis H5**. Sensation seeking also mediated this relationship (*ES* = −0.016, 95% *CI* [−0.023, −0.009]), supporting **hypothesis H6**. The chain mediating effect of self-efficacy and sensation seeking between water safety knowledge and high-risk swimming behaviors was significant (*ES* = 0.003, 95% *CI* [0.001, 0.005]), supporting **hypothesis H7**.

The results of the mediation analysis are presented in Table 6 and Figure 2.

**Table 6:** Results of bootstrap analysis.

Path	Effect	Boot *SE*	Bootstrapping 95% *CI*	
			LLCI	ULCI
WSK → RP → HSB	0.003	0.002	−0.001	0.008
WSK → RA → HSB	−0.035	0.007	−0.050	−0.020
WSK → SE → HSB	0.002	0.001	0.00001	0.005
WSK → SS → HSB	−0.016	0.003	−0.023	−0.009
WSK → RP → RA → HSB	0.002	0.001	−0.001	0.004
WSK → SE → SS → HSB	0.003	0.001	0.001	0.005
Direct Effect	0.006	0.010	−0.014	0.026
Total Effect	−0.037	0.012	−0.059	−0.009

The sample size for Bootstrap resampling is set to 5000; the 95% confidence interval (*CI*) for Bootstrapping is calculated using the non-parametric bias-corrected percentile method. Effect sizes and interval limits are reported to three decimal places, except the lower limit of the *WSK*→*SE*→*HSB* interval, which is reported to five decimal places because it rounds to 0.000 at three decimals.

### 3.4 Fuzzy-set qualitative comparative analysis

#### 3.4.1 Analysis of necessity

We conducted a necessity analysis on water safety knowledge, risk perception, risk attitude, swimming self-efficacy, and sensation seeking. Results appear in Table 7. The highest consistency for any single condition in predicting high-risk swimming behaviors was 0.847, and none exceeded 0.9 (Fainshmidt et al., 2020). Thus, no single condition can independently account for high-risk swimming behaviors. For non–high-risk behaviors, two conditions approached or exceeded the conventional 0.90 necessity threshold, with an asymmetric distribution: ~*RA* (0.974), indicating a cautious attitude, and ~*SS* (0.915), indicating low-level sensation seeking. In set-theoretic terms, the absence of a risk-tolerant attitude and of elevated sensation seeking is almost always observed among cases without the outcome. This finding corroborates, from a configurational perspective, the dominant roles of risk attitude and sensation seeking evident in the mediation analysis.

**Table 7:** Results of necessary condition analysis.

Variable	HSB		~HSB	
	Consistency	Coverage	Consistency	Coverage
WSK	0.673	0.597	0.675	0.681
~ WSK	0.641	0.634	0.600	0.676
RP	0.675	0.611	0.694	0.715
~ RP	0.685	0.663	0.623	0.686
RA	0.317	0.916	0.170	0.560
~ RA	0.847	0.473	0.974	0.619
SE	0.605	0.674	0.462	0.586
~ SE	0.629	0.507	0.743	0.682
SS	0.419	0.813	0.247	0.545
~ SS	0.765	0.471	0.915	0.642

~ represents the logical operation of complement.

#### 3.4.2 Configuration analysis

As detailed in the Methods, the case frequency threshold was set at 3, the raw consistency threshold at 0.80, and the PRI consistency threshold at 0.65; 27 of the 32 logically possible truth-table rows met the frequency threshold and covered 99.49% of the 1,573 cases. Boolean minimization of the five conditions yielded seven configurations associated with high-risk swimming behaviors (see Table 8).

**Table 8:** Results of configurational analysis of high-risk swimming behaviors.

Conditions	Configuration 1	Configuration 2	Configuration 3	Configuration 4	Configuration 5	Configuration 6	Configuration 7
WSK	⊗		⊗	⊗		●	●
RP		⊗	•			⊗	
RA	●	⊗	●		●	●	⊗
SE	⊗			●	●	⊗	⊗
SS		●		●	●		●
Raw coverage	0.200	0.271	0.230	0.280	0.232	0.190	0.212
Unique coverage	0.004	0.024	0.002	0.017	0.018	0.009	0.008
Consistency	0.953	0.936	0.949	0.927	0.960	0.964	0.936
Solution coverage	0.431						
Solution consistency	0.887						

● indicates a core present condition; • indicates a peripheral present condition; ⊗ indicates a core absent condition; ⊗ indicates a peripheral absent condition. A blank indicates that the condition is optional. The same applies hereinafter.

Table 8 presents the configurations, showing that high-risk swimming behaviors do not depend on a single factor but arise from complex pathways formed by different combinations of five antecedent variables. The overall solution consistency for high-risk swimming behaviors is 0.887, and the solution coverage is 0.431. In the overall configuration analysis, we foreground the two configurations in which water safety knowledge is a core present condition.

Because the research question concerns the knowledge and behavior relationship, the Discussion foregrounds the configurations in which water safety knowledge appears as a core present condition, namely Configurations 6 and 7, the only two solutions in which knowledge is core and present. The remaining configurations are summarized here. Configuration 1 combines a core present risk-tolerant attitude with the core absence of knowledge and self-efficacy. Configuration 2 combines core present sensation seeking with the core absence of risk perception and a cautious attitude. Configuration 3 combines core present risk perception and a risk-tolerant attitude with the core absence of knowledge. Configuration 4 combines core present self-efficacy and sensation seeking with the core absence of knowledge. Configuration 5 combines a risk-tolerant attitude, self-efficacy, and sensation seeking as jointly core present conditions. Configurations 4 and 5, which share core present self-efficacy and sensation seeking, are interpreted jointly in the Discussion as the “Ineffective efficacy—impulsive penetration” pattern. The low risk tolerance and low sensation seeking recur as core conditions, consistent with the necessity analysis in which the absence of a risk-tolerant attitude (consistency 0.974) and the absence of sensation seeking (consistency 0.915) approach necessity for non-high-risk behaviors.

(1) “Disconnect between knowledge and action—attitude deviation type”: In Configuration 6, water safety knowledge appears as a core condition and, when combined with a risk-tolerant, adventurous attitude, together with core-absent risk perception and core-absent self-efficacy (sensation seeking is optional in this configuration), forms the antecedent conditions for high-risk swimming behaviors. The configuration's consistency is 0.964, indicating that the configuration is sufficient for the outcome in the large majority of covered cases. Its raw coverage is 0.190, meaning this configuration accounts for 19.0% of membership in the outcome; unique coverage is 0.009. Although water safety knowledge is central, sensation seeking plays no defining role in this configuration; individuals who pay little attention to risks and adopt adventurous attitudes can arrive at the outcome without an impulsivity profile. Children may recognize danger yet still favor adventurous conduct. This pattern shows that knowledge is not necessarily accompanied by protective behavior; in this configuration, knowledge can co-occur with risky action, a pattern consistent with a disconnect between knowledge and action.

(2) “Disconnect between knowledge and action—impulse breakthrough type”: In Configuration 7, water safety knowledge and a high sensation-seeking tendency constitute core conditions that, together with a safety-oriented attitude and low efficacy belief, are associated with high-risk swimming behaviors. The configuration solution has a consistency of 0.936, indicating that the configuration is sufficient for the outcome in the large majority of covered cases. Its raw coverage is 0.212, so this configuration accounts for 21.2% of membership in the outcome; unique coverage is 0.008. Although children possess sufficient knowledge to adopt a cautious stance toward recognized hazards, their elevated sensation seeking coincides with continued risky engagement. Consequently, children may acknowledge the danger, yet their desire for stimulation is associated with continued engagement in risky behaviors.

### 3.5 Robustness test

Because fsQCA results can be sensitive to parameter choices, we tested robustness by varying two thresholds. Raising the case-frequency threshold from 3 to 4 left 99.30% of cases with the same configurational assignments, and increasing the consistency threshold from 0.80 to 0.85 produced comparable outcomes (Skaaning, 2011). For high-risk swimming behaviors, the robustness tests indicate that the overall solution configuration and the core subset of the original pathway remain unchanged. Specifically, solution coverage changed only from 0.431 to 0.429, while overall consistency rose from 0.887 to 0.890. Recalibrating with the 90th, 50th, and 10th percentile anchors and rerunning the analysis left the core configurations unchanged; this recalibration shifts the anchors for water safety knowledge, risk perception, self-efficacy, and high-risk swimming behaviors, whereas the anchors for risk attitude and sensation seeking coincide at the scale minimum (Table 2) and are unaffected by it. These findings indicate that the configurational results are robust.

Integration of symmetric and asymmetric findings. The two analytical strategies answer different questions, and their results align in four ways. First, the configurational results corroborate the mediation findings for risk attitude and sensation seeking. The two largest indirect paths run through risk attitude (*ES* = −0.035) and sensation seeking (*ES* = −0.016), and the same two conditions recur as core conditions across the high-risk configurations, while their absence approaches necessity for non-high-risk behaviors (consistencies 0.974 and 0.915). Second, the configurations qualify the self-efficacy paths. The positive indirect effects through self-efficacy (*ES* = 0.002) and through the self-efficacy to sensation seeking chain (*ES* = 0.003) indicate that knowledge is associated with higher efficacy beliefs that can coincide with risk-taking; the configurations show that high self-efficacy appears in high-risk configurations only in combination with core present sensation seeking (Configurations 4 and 5), never as a standalone condition. Third, the configurational results reconcile the apparently contradictory role of knowledge. The regression model found no direct association between knowledge and high-risk behaviors (β = 0.006, *p* > 0.05), yet knowledge is a core present condition in Configurations 6 and 7. In knowledge is neither necessary nor sufficient on its own but functions as an INUS condition, an insufficient but non-redundant part of two sufficient combinations. Both analyses therefore converge on the same conclusion: knowledge is associated with high-risk swimming behavior only through, and only in combination with, psychological conditions. Fourth, risk perception plays no independent role in either analysis; its indirect path is not significant (*ES* = 0.003, 95% *CI* [−0.001, 0.008]), and it appears as a core present condition in only one of the seven configurations (Configuration 3).

## 4 Discussion

The knowledge–belief–behavior transformation mechanism. Although the two mediating channels operate in parallel at the model level, each channel instantiates a sequential transformation chain through which cognitive information is converted into behavioral decisions. In the cognition–emotion channel, water safety knowledge first updates threat beliefs: children appraise the susceptibility to and severity of water hazards, a belief-updating function that risk perception serves in the Health Belief Model. These appraisals are then deliberatively weighed—through the slow, analytical System 2 rather than the intuitive System 1 (Kahneman et al., 2011)—and consolidated into a relatively stable attitudinal disposition toward risk, which in turn guides behavior (Slovic and Peters, 2006; Slovic et al., 2013). Risk perception and risk attitude were conceptualized as successive stages of one transformation sequence rather than two independent parallel mediators: the former identifies the risk problem, and the latter converts that identification into an action tendency. The chained path through both mediators was not supported in the present data, so this sequential account remains a theoretical proposal that the current results only partially inform. Critically, the direction of belief updating is not fixed. The negative association between water safety knowledge and risk perception observed here indicates that greater knowledge does not uniformly heighten perceived threat; under certain psychological conditions it co-occurs with lower threat appraisal. This non-monotonicity is consistent with the risk-as-feelings account, in which cognitive evaluations and affective reactions jointly—and sometimes divergently—shape decisions (Loewenstein et al., 2001), and with the risk perception paradox documented in natural-hazard research, where heightened risk perception does not necessarily translate into protective behavior (Wachinger et al., 2013). The configurational results specify where the transformation succeeds or fails: in the “disconnect between knowledge and action—attitude deviation” configuration the chain breaks at the attitude-formation stage, whereas in the “disconnect between knowledge and action—impulse breakthrough” configuration it is overridden at the decision stage by sensation seeking. This sequential, belief-mediated transformation is also consistent with behavioral-acceptance research across educational, transportation, and emerging-mobility contexts, where risk-related information shapes behavioral intention mainly indirectly, through belief-based constructs such as perceived usefulness, trust, and self-efficacy, rather than directly (Hu et al., 2024, 2025, 2026).

Drowning prevention research has repeatedly documented a gap between what children know and what they do in the water, but it has largely treated that gap as a failure of knowledge transmission. Our results suggest a different structure. First, both KAP and HBM treat knowledge and efficacy beliefs as uniformly protective resources. In this capability-dependent risk domain, that monotonicity fails at the model level: water safety knowledge, operationalized here as self-assessed mastery, had no direct association with high-risk swimming (β = 0.006, *ns*), and its indirect associations split into opposing channels, protective through risk attitude (*ES* = −0.035) and sensation seeking (*ES* = −0.016), risk-facilitating through self-efficacy (*ES* = 0.002) and the self-efficacy to sensation seeking chain (*ES* = 0.003). The knowledge–action gap is therefore structured as competition between indirect channels, not as failed transmission, a pattern neither *KAP*'s linear chain nor HBM's additive beliefs can represent. Second, the configurational analysis specifies when knowledge matters. Knowledge was an INUS condition, neither necessary nor sufficient on its own, but a non-redundant ingredient of two sufficient configurations (Configurations 6 and 7), while equifinal configurations that bypass knowledge entirely are also sufficient for the outcome (Configurations 1 to 5). With respect to prior work, the attitude and sensation seeking paths confirm the findings of Luo et al. (2024) and Shi et al. (2025) within an integrated model, while the risk-facilitating efficacy channel and the configurational structure specify where the standard accounts need qualification. The contribution to drowning prevention theory is thus not a new list of predictors but a mechanism-level account of why knowledge interventions so often disappoint: knowledge is associated with behavior only through, and in combination with, psychological conditions, and one of those channels can run in the risk-facilitating direction.

### 4.1 Relationship between water safety knowledge and high-risk swimming behaviors

**Research hypothesis H1** stated that water safety knowledge would negatively predict high-risk swimming behaviors among school-age children. This hypothesis was not supported. According to the Knowledge, Attitude, Practice (*KAP*) theory, knowledge alone cannot facilitate behavioral change without psychological mediating factors such as attitude, belief, and efficacy assessment. In this study, safety knowledge was not directly associated with fewer high-risk behaviors, underscoring the persistent “knowledge-action gap.” School-age children who systematically acquire water safety knowledge—such as recognizing warning signs in hazardous waters, understanding emergency response measures for drowning, and adhering to protective procedures—must internalize this knowledge by accurately perceiving swimming risks to avoid behaviors like swimming alone or entering unfamiliar waters (Alves, 2024). Knowledge was associated with behavior through psychological expectations, risk appraisal, and safety attitudes (Moran, 2007). Consequently, water safety knowledge provides a cognitive foundation for hazard identification and serves as a prerequisite for informed behavioral choices through various psychological mediating factors.

### 4.2 Mediating effect and chain mediating effect of risk perception and risk attitude

Water safety knowledge was negatively associated with risk perception (β = −0.147), and the indirect estimate through risk perception was positive but not significant (*ES* = 0.003, 95% *CI* [−0.001, 0.008]); **H2** was therefore not supported. We do not interpret the negative association between knowledge and risk perception as evidence of distorted risk processing, because our design includes no benchmark against which distortion could be identified. At least two accounts are compatible with the data. Knowledge may calibrate threat appraisal toward less alarmed and potentially more accurate estimates, or knowledge may co-occur with a dampening of perceived risk that may or may not be warranted in some children. Distinguishing calibration from distortion requires objective risk information and direct measures of the candidate mechanisms, which we did not collect. We therefore offer these accounts as hypotheses for future research rather than as conclusions.

**Research hypothesis H3**: “Risk attitude plays a complete mediating role between water safety knowledge and high-risk swimming behaviors.” The results indicate that attitude functions as a stable, moderate bridge that is associated with the translation of knowledge into behavior. Water safety knowledge was not directly associated with behavior; rather, it was associated with attitude, plausibly through a subjective trade-off between expected benefits (stimulating experience, peer recognition) and expected losses (drowning risk, physical injury), and was then indirectly associated with fewer high-risk swimming behaviors among school-age children. One candidate account is that emotional safety materials (for example, drowning cases and risk images) create an associative link of “high-risk situation–negative emotion” in long-term memory, which may allow emotional heuristics to rapidly activate the attitude that risks are not worth taking. This early activation may interrupt decision-making when the impulsive system initiates action, a candidate pathway by which emotional attitudes may counter impulsive drives (Steinberg et al., 2008; Slovic and Peters, 2006).

**Research hypothesis H4** stated that risk perception and risk attitude would jointly form a chained mediation pathway between water safety knowledge and high-risk swimming behaviors. This hypothesis was not supported. Cognitive judgment of risk perception reflects processed information about risk probability and severity, while emotional evaluation of risk attitude denotes the affective disposition toward risk-taking (Slovic et al., 2013; Savadori and Lauriola, 2022). In this study, the indirect estimate through risk perception was positive but not significant (*ES* = 0.003, 95% *CI* [−0.001, 0.008]), whereas the indirect path through risk attitude was significant and negative (*ES* = −0.035, 95% *CI* [−0.050, −0.020]). The chained indirect estimate was not significant (*ES* = 0.002, 95% *CI* [−0.001, 0.004]). The framing effect offers one candidate account: the negative association between safety knowledge and risk perception may create a “relatively safe” cognitive frame that conflicts with the “relatively dangerous” warning frame required to elicit a cautious risk attitude, which could undermine the transmission from risk perception to risk attitude (Lacombe et al., 2025). The absence of chain mediation thus supports the view that, within the integrated KAP-HBM framework, knowledge was associated with behavior through several independent paths.

### 4.3 Mediating effects and chain mediating effects of self-efficacy and sensation seeking

**Research hypothesis H5** states that “Self-efficacy provides an independent mediating path between water safety knowledge and high-risk swimming behaviors.” The findings support the central claim of the Health Belief Model that self-efficacy links intention and action in health behavior change (Love-Smith et al., 2022). That model proposes that individuals' beliefs about their health condition convert cognition into willingness to adopt health behaviors (Jones et al., 2015). In this study, school-age children gained confidence in their swimming skills as they acquired knowledge. Paradoxically, higher confidence was associated with more, not less, high-risk behavior in this sample. This pattern is consistent with the view that self-efficacy can act as a “double-edged sword” requiring careful management (Tan et al., 2025). One candidate explanation is that confidence may outpace actual ability when children face water hazards; rather than promoting caution, higher efficacy beliefs were associated with more high-risk swimming in this sample (Shulman et al., 2016).

**Research hypothesis H6** states that “sensation seeking provides an independent mediating path between water safety knowledge and high-risk swimming behaviors.” The study integrates personality traits into health integration theory to explain why children with safety knowledge may nonetheless engage in risky behaviors. Higher water safety knowledge was associated with lower sensation seeking, and lower sensation seeking was in turn associated with fewer high-risk swimming behaviors. This pathway rests on the theoretical premise that sensation seeking exhibits both stability and plasticity (Lynne-Landsman et al., 2011). The plasticity of sensation seeking offers a practical target for interventions that seek to translate knowledge into behavior. Therefore, future drowning-prevention programs should include training modules that directly address sensation seeking—such as mindfulness and impulse-control training—to shift interventions from “knowledge dissemination” toward “impulse management” (Khabaznoori and Azad, 2024; Najafi Chaleshtori et al., 2022).

The positive indirect paths through self-efficacy (*ES* = 0.002) and through the self-efficacy to sensation seeking chain (*ES* = 0.003) indicate that higher self-rated swimming confidence is associated with more high-risk swimming, both directly and in combination with sensation seeking. Our scale measures confidence in completing progressively longer swims (Theodorakis, 1995); it measures perceived capability, not overconfidence, which would require an objective ability benchmark. We therefore describe the pattern as an association between self-rated confidence and risk-taking, and we present the efficacy-miscalibration account proposed in prior work (Tan et al., 2025) as one candidate explanation among several. An equally plausible account is that more capable swimmers simply attempt more. Direct tests of these accounts require objective measures of swimming ability.

### 4.4 Influence of combinations of different conditions on high-risk swimming behaviors among school-age children in China

(1) “Disconnect between knowledge and action—attitude deviation type”: This pattern refers to children who, without sensation seeking as a defining condition, show a clear gap between risk knowledge and risky behavior, typified by “knowing the risk but still taking it.” One candidate account is that, at their current cognitive stage, children memorize safety information as static labels rather than transforming it into principles that guide action. When risk cues are limited to written warnings and children do not receive contextualized training in risk reasoning, the cognitive binding of that knowledge weakens and it was not accompanied by a prudent attitude (Bulterman et al., 2025). This configuration supplements the non-protective side of the WSK → RA → HSB indirect path. If children have high knowledge but also a solitary propensity for risk-taking and greater tolerance for potential harm, their knowledge reserves may not restrain high-risk swimming, resulting in a structural decoupling of cognition and action. The attitude channel is further corroborated by the near-necessary cautious attitude observed for non–high-risk swimming behaviors (~RA = 0.974), which underscores the role of attitude and belief as a psychological mediator in KAP theory.

(2) “Disconnect between knowledge and action—impulse breakthrough type”: This pattern occurs when children have water-safety knowledge and adopt a cautious attitude toward drowning, yet developmental accounts in which the reward system matures earlier than executive control offer one candidate explanation for such impulsive behavior (Steinberg et al., 2008). The appeal of exploring unfamiliar water and the strong visual and tactile stimulation of water provide compelling incentives for water-related activity. Without accurate risk perception, children may misjudge the deceptively flat appearance of shallow areas and seek pleasurable “shallow-water stimulation.” This configuration complements the WSK → SS → HSB indirect path in reverse, because the typical protective association of knowledge breaks down for high sensation-seekers. In such moments, knowledge is not accompanied by immediate avoidance of high-risk behavior, a disconnect between knowledge and action. The high consistency of low sensation seeking for non-high-risk swimming behaviors (~*SS* = 0.915) further corroborates its near-necessary role on the protective side.

(3) “Ineffective efficacy — impulsive penetration type”: Configurations 4 and 5 indicate that when children hold relatively strong efficacy beliefs and high sensation-seeking tendencies, their confidence in exploring unfamiliar waters and the presence of visual and tactile cues become the primary psychological conditions associated with water-related behavior. The main causes are as follows. A pronounced risk-taking tendency, efficacy beliefs that may exceed actual ability (a candidate account), and elevated sensation-seeking are characteristic of preadolescents. Even in hazardous environments, the appeal of the underwater setting and the mismatch between perceived and actual ability may impair rational threat appraisal and divert attention from safety, a pattern associated with elevated drowning risk. This configuration thus illustrates how the HBM pathway can be overridden at the decision stage, consistent with the positive WSK → SE → SS → HSB indirect path.

Generalizability to broader school-aged populations. The sample was drawn from four primary schools in one province and showed little age variation (*M* = 11.31, *SD* = 0.46 years, grades five and six), so the estimates apply directly to older primary-school children in central China, and we have scoped the population statements accordingly. Whether the mechanisms extend to other ages deserves separate comment, because the developmental literature that motivates this study predicts that they should not operate identically across childhood. The attitude channel, in which knowledge is associated with behavior through risk attitude, reflects a general knowledge, attitude, and behavior sequence documented across age groups and is the most plausible candidate for age robustness. The impulse channel is likely to be age sensitive: sensation seeking rises into mid-adolescence before declining, while cognitive control matures through the same period (Steinberg et al., 2008; Shulman et al., 2016). The self-efficacy to sensation seeking pathway observed here may therefore be weaker in younger children and stronger in adolescents than in our preadolescent sample. This developmental window is precisely why we studied this age group: preadolescence precedes the mid-adolescent surge in sensation seeking, and it is the stage at which systematic water-safety instruction occurs in Chinese primary schools. The configurational findings are likewise sample-bound, and different age compositions may yield different sufficient combinations. Testing the developmental moderation of these pathways requires age-heterogeneous or longitudinal samples, ideally with pubertal measures alongside chronological age, and samples drawn from multiple regions.

The findings carry several suggestions for drowning prevention practice, which we offer together with their caveats. (1) The two largest indirect paths run through risk attitude and sensation seeking, and low risk tolerance and low sensation seeking approach necessity for non-high-risk behaviors; this pattern suggests that knowledge instruction alone is unlikely to be sufficient and that attitude-focused and impulse-management components are plausible complements. (2) The configurational results indicate that high-risk behavior arises under distinct profiles, including an attitude-deviation profile and an impulsive-breakthrough profile, so programs may benefit from screening-based tailoring rather than uniform curricula. (3) The positive association between self-rated swimming confidence and risk-taking suggests possible value in helping children calibrate confidence against actual ability, for example through supervised skills practice with feedback. These suggestions are tempered by four considerations. The evidence is cross-sectional and associational, so the recommended targets are hypothesis-generating rather than evaluated intervention components. Some indirect paths are small, and their practical importance awaits replication and intervention testing. The sample represents older primary-school children in central China, and program design for other ages and regions should not assume that these estimates transfer. Finally, components such as impulse-control training have supporting evidence mainly outside the water-safety domain, and their effectiveness for drowning prevention remains to be tested.

## 5 Conclusions

This study examined the psychological mechanisms and configurational patterns associated with high-risk swimming behaviors among older primary-school children in central China. (1) Water safety knowledge was associated with high-risk swimming behaviors only indirectly, through several psychological mediators. This pattern is consistent with the view that knowledge functions as a cognitive foundation whose relation to behavior operates through expectations, attitudes, and beliefs. (2) Along the cognitive–affective pathway, higher knowledge was associated with a less risk-tolerant attitude, which in turn was associated with fewer high-risk behaviors. One plausible interpretation is that knowledge is linked to attitude through a weighing of anticipated gains and losses and through affective associations between high-risk situations and negative emotions. (3) The motivation–impulse pathway ran in both directions. Higher knowledge was associated with lower sensation seeking, and lower sensation seeking was associated with fewer high-risk behaviors. In contrast, the indirect paths through self-efficacy and through the self-efficacy to sensation seeking chain were positive, that is, associated with more high-risk behaviors. Because the study measured perceived swimming self-efficacy rather than objective swimming ability, these positive paths are consistent with, but cannot demonstrate, an overconfidence or belief-miscalibration account. (4) The configurational analysis corroborated the non-linear and equifinal character of these associations, and under particular psychological combinations higher knowledge co-occurred with more high-risk behavior. Taken together, the results suggest that drowning-prevention programs may benefit from pairing knowledge transmission with attitude calibration and impulse management.

## 6 Limitations

Several limitations qualify the findings. First, the design is cross-sectional and single-wave, so the temporal ordering of the mediators and the outcome cannot be established and the paths are associational; longitudinal and experimental designs are needed to test the proposed sequence. Second, all constructs were self-reported by the same respondents at one time point, which raises the possibility of common-method variance even though the single-factor diagnostic was below the conventional threshold. Third, water safety knowledge was assessed by self-report and reflects perceived knowledge mastery rather than objectively verified knowledge; future studies should combine self-report scales with objective knowledge tests. Fourth, the design included no objective benchmarks for risk exposure or swimming ability, so the data cannot identify cognitive distortion, overconfidence, or efficacy miscalibration, and such mechanisms are offered only as candidate explanations. Fifth, the risk perception scale showed comparatively weak measurement properties (α = 0.858, *AVE* = 0.305), and the six-factor measurement model showed modest incremental fit (*CFI* = 0.863, *TLI* = 0.857); both may contribute to its null indirect path. Sixth, the mediation analysis used observed composite scores rather than latent variables, which treats measurement error as absent. Measurement error attenuates path estimates, and because reliability differed across constructs, attenuation is unequal across paths, so comparisons of indirect-effect magnitudes between mediators should be read with caution. The regression equations were also estimated separately and do not model correlated disturbances among the mediators. A latent structural equation model would relax these constraints and could yield somewhat different point estimates. As a robustness check, we re-estimated the full serial mediation model as a latent CB-SEM. The direction and significance of the specific indirect effects were unchanged, with one exception: the near-zero positive indirect path through risk perception (*WSK*→*RP*→*HSB*), non-significant under PROCESS (*ES* = 0.003, 95% *CI* [−0.001, 0.008]), reached marginal significance under SEM (*ES* = 0.004, 95% *CI* [0.000, 0.008]). Both point estimates are negligible in magnitude, so the substantive interpretation is unaffected; the configurational analysis does not depend on this estimation choice. Seventh, the configurational analysis has three boundaries. The calibration anchors were defined by sample percentiles, so membership scores are sample-relative and replications in other populations should recalibrate rather than reuse these anchors. The solution coverage of 0.431 indicates that the seven configurations account for about 43 percent of membership in the outcome, and the remaining membership is distributed across combinations outside the model, which is consistent with equifinality but also means that conditions this study did not measure may matter. The unique coverages were small (0.002 to 0.024), so the configurations are best read as several interchangeable sufficient combinations rather than as distinct subgroups. Eighth, the sample came from four schools in one province and covered only grades five and six with limited age variation, and students were nested within schools; the estimates apply directly to older primary-school children in central China, and replication in other regions and age groups is needed before the results can be generalized further.

## Data Availability

The original contributions presented in the study are included in the article/Supplementary material, further inquiries can be directed to the corresponding author.
